# Assessment of atherosclerotic plaque burden: comparison of AI-QCT versus SIS, CAC, visual and CAD-RADS stenosis categories

**DOI:** 10.1007/s10554-024-03087-x

**Published:** 2024-04-17

**Authors:** Hufsa Khan, Kopal Bansal, William F. Griffin, Catherine Cantlay, Alfateh Sidahmed, Nick S. Nurmohamed, Robert K. Zeman, Richard J. Katz, Ron Blankstein, James P. Earls, Andrew D. Choi

**Affiliations:** 1 Division of Cardiology, The George Washington University School of Medicine, Washington, DC USA; 2 Department of Radiology, The George Washington University School of Medicine, Washington, DC USA; 3 Department of Radiology, University of North Carolina, Chapel Hill, NC USA; 4 Cardiovascular Division and Department of Radiology, Brigham and Women’s Hospital, Boston, MA USA; 5 Cleerly Healthcare, Denver, CO USA

**Keywords:** CCTA, AI, Atherosclerosis, SIS, CACS, CAD-RADS, Plaque burden

## Abstract

This study assesses the agreement of Artificial Intelligence-Quantitative Computed Tomography (AI-QCT) with qualitative approaches to atherosclerotic disease burden codified in the multisociety 2022 CAD-RADS 2.0 Expert Consensus. 105 patients who underwent cardiac computed tomography angiography (CCTA) for chest pain were evaluated by a blinded core laboratory through FDA-cleared software (Cleerly, Denver, CO) that performs AI-QCT through artificial intelligence, analyzing factors such as % stenosis, plaque volume, and plaque composition. AI-QCT plaque volume was then staged by recently validated prognostic thresholds, and compared with CAD-RADS 2.0 clinical methods of plaque evaluation (segment involvement score (SIS), coronary artery calcium score (CACS), visual assessment, and CAD-RADS percent (%) stenosis) by expert consensus blinded to the AI-QCT core lab reads. Average age of subjects were 59 ± 11 years; 44% women, with 50% of patients at CAD-RADS 1–2 and 21% at CAD-RADS 3 and above by expert consensus. AI-QCT quantitative plaque burden staging had excellent agreement of 93% (k = 0.87 95% CI: 0.79–0.96) with SIS. There was moderate agreement between AI-QCT quantitative plaque volume and categories of visual assessment (64.4%; k = 0.488 [0.38–0.60]), and CACS (66.3%; k = 0.488 [0.36–0.61]). Agreement between AI-QCT plaque volume stage and CAD-RADS % stenosis category was also moderate. There was discordance at small plaque volumes. With ongoing validation, these results demonstrate a potential for AI-QCT as a rapid, reproducible approach to quantify total plaque burden.

## Introduction

Multiple studies have established that increasing atherosclerotic plaque burden identified by cardiac computed tomography angiography (CCTA) is an effective discriminator of cardiovascular events [1–6]. Creating a framework to translate these findings to clinical practice is of recent interest with the publication of 2022 Coronary Artery Disease-Reporting and Data System: Expert Consensus of the Society of Cardiovascular Computed Tomography/American College of Radiology/American College of Cardiology (CAD-RADS 2.0) [7]. CAD-RADS 2.0 provides a standardized approach to CCTA reporting to easily communicate findings, guide appropriate downstream testing and enable evidence based decision making. This expert consensus document allows for plaque burden assessment via coronary artery calcium score (CACS), segment involvement score (SIS), or a visual assessment of the overall burden of CAD. However, these approaches have limitations. SIS and visual assessment may include a subjective assessment and may be prone to inter-reader variability while CACS does not account for non-calcified plaque, and is not routinely performed with every CCTA study.

Recently, advances in data science have allowed for rapid assessment of coronary atherosclerosis burden [8–10]. Artificial intelligence guided quantitative computed tomography (AI-QCT) may allow for a validated approach to cardiovascular risk prediction beyond current expert consensus approaches [9]. This study aims to assess the agreement of AI-QCT with qualitative approaches to atherosclerotic disease burden codified in CAD-RADS 2.0, including SIS, CACS, visual assessment, and CAD-RADS percent (%) stenosis.

## Methods

### Subjects

We evaluated data from 105 consecutive patients as a convenience sample from the CLARIFY (CT Evaluation by Artificial Intelligence for Atherosclerosis, Stenosis and Vascular Morphology) study of patients undergoing CCTA for stable and acute chest pain imaged at our local institution that included both contrast and non-contrast CT scans [9]. The study was approved by the George Washington University Institutional Review Board with a waiver of patient consent. This study was investigator-initiated. We included all CLARIFY patients at our site for which we had available data. We identified consecutive patients undergoing CCTA for acute and stable chest pain. CLARIFY excluded exams with incomplete data, significant artifacts, poor enhancement, stents or bypass grafts.

## CT imaging protocols

CCTA were performed using a 128-dual source Siemens FLASH (Siemens Healthcare, Erlangen, Germany). CCTA was performed in accordance with guidelines from the Society of Cardiovascular Computed Tomography (SCCT) [11]. Acquisition techniques included prospective and retrospective gating based upon institutional protocols followed by iterative reconstruction. Patients received beta blockade, nitroglycerin, and iodinated contrast in accordance with institutional and Society of Cardiovascular Computed Tomography guidelines. Exams were reconstructed in 5–10% increments. We excluded exams with incomplete data, significant artifacts, poor enhancement, stents or bypass grafts.

## AI-based segmentation, stenosis quantification, plaque characterization

**Fig. 1:**
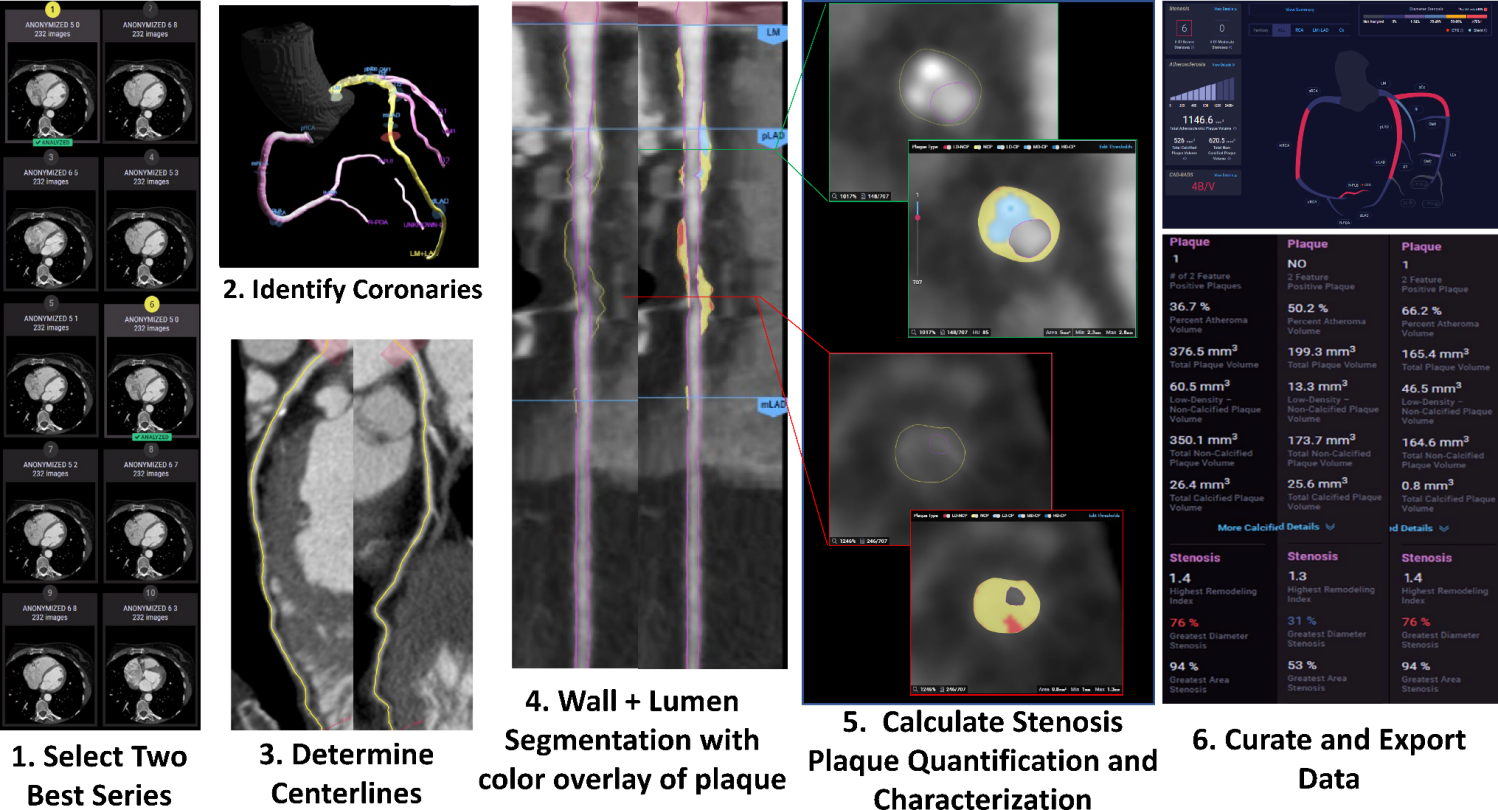
Artificial Intelligence-Quantitative Computed Tomography (AI-QCT) Methodology. As shown above, the AI-QCT (1) selects the two best series, then (2) identifies and labels the coronary arteries, and (3) determines the centerlines. Then, (4) there is automated segmentation of the coronary artery wall and lumen, after which it applies a color overlay to the plaque. Next, (5) there is calculation of % stenosis, quantification of plaque volume and assessment of plaque characterization. (6) After a quality assurance review, the data is exported and curated in a graphic interface. Please note that specific values are shown for illustrative purposes

As previously published, AI-guided approach to coronary CTA interpretation (Fig. 1) was performed using a FDA-cleared software service (Cleerly Labs, Cleerly, Inc, Denver CO) CCTA that performs automated analyses using a series of validated convolutional neural network models (including VGG19 network, 3D U-Net, and VGG Network Variant that produce an identical result each iteration) for image quality assessment, coronary segmentation and labeling, lumen wall evaluation and vessel contour determination, and plaque characterization with a previously published time for mean AI analysis of 9.7 ± 3.2 min [9]. Quantitative atherosclerosis characterization was performed for every coronary artery and its branches. Coronary segments with a diameter ≥ 2 mm were included in the analysis using the modified 18-segment SCCT model [12]. Each segment was evaluated for the presence or absence of coronary atherosclerosis, defined as any tissue structure > 1 mm^3^ within the coronary artery wall that was differentiated from the surrounding epicardial tissue, epicardial fat, or the vessel lumen itself.

Total plaque volume (TPV) (mm^3^) was calculated as the sum of all coronary plaque present, while non-calcified plaque volume was defined as plaque volume between 30 to 350 HU (1,10).

Applying a recently proposed staging system of plaque quantification by Min, et al., AI-QCT TPV was categorized as Stage Zero (0–10 mm^3^), Stage 1 Mild (11–250 mm^3^), Stage 2 Moderate (250–750 mm^3^), and Stage 3 Severe (> 750 mm^3^) [13]. As this is a novel technique, the category in this study of > 0-10 mm^3^ was included within the zero category to account for variability in inter-scan plaque volume assessment by AI-QCT and the spatial resolution of quantitative analysis of 2–3 mm^3^.

Standard visual methods of overall plaque quantification including segment involvement score (SIS), coronary artery calcium score, visual subjective assessment, and CAD-RADS were additionally performed by the consensus of an Independent Practitioner (IP) and Advanced Practitioner (AP) blinded to the AI-QCT following criteria included in CAD-RADS 2.0 [14]. SIS was determined by visual assessment and summed based on presence or absence of plaque with one point assigned on a per segment basis (7). SIS was further categorized as none (0 segments), mild (1–4 segments), moderate (5–7 segments) and severe (≥ 8 segments). The subjective visual plaque estimate was graded as none, mild (1–2 vessels with mild plaque), moderate (1–2 vessels with moderate or 3 vessels with mild), severe (3 vessels with moderate, 1 vessels with severe) or extensive (2–3 vessels with severe). CACS was calculated on non-contrast gated CT using the Agatston method, and was categorized as none (0), mild (1–100), moderate (101–300), severe (> 300). CAD-RADS % stenosis category (none (0, 0%), mild (1–2, 1–49%), moderate (3, 50–69%), and severe (4–5, 70–99%, 100%) was scored based upon the maximum percent stenosis in accordance with the guidance document [15]. When there was 0% diameter stenosis but plaque was identified, the CAD-RADS stenosis category was recorded as 1 per CAD-RADS 2.0 guidelines.

## Statistical analysis

All statistical analyses were performed using MedCalc (MedCalc Software Ltd, Ostend, Belgium). Continuous data are reported as mean ± SD, median (IQR 25th-75th percentile) and categorical variables are presented as absolute numbers with corresponding frequencies. via the simple kappa statistic was calculated and further visualized via contingency tables [16]. Differences in quantified plaque volume was assessed by Mann–Whitney U testing.

## Results

This study included 105 patients which had a mean age of 59 ± 11 years; 44% of patients were women **(**Table 1**)**. Sixty-two patients (60%) had hyperlipidemia, 34 (33%) had diabetes while 55 (54%) were current or past smokers. The median CACS score was 23 by the Agatston method (IQR 25–75: 0–106). 30 subjects (29%) were CAD-RADS 0, 53 (50%) were CAD-RADS 1–2, and 22 (21%) were at least CAD-RADS 3 by expert consensus.

**Table 1:** Demographic data

(N = 105)		
Variable	N	Percentage
Mean Age ± SD (YEARS)	59 ± 11	
Race: Caucasian	31	30%
Race: African American	52	51%
Female Sex	46	44%
Male Sex	59	56%
BMI ± SD	30 ± 6	
Hypertension	65	64%
Hyperlipidemia	62	59%
Diabetes Mellitus	34	32%
Current Smoker	35	33%
Past Smoker	20	19%
Family History of CAD	24	24%
Statin Therapy	43	4%
Antiplatelet Therapy	26	25%
Beta Blocker Therapy	21	21%
Coronary Artery Calcium Score by Agatston method: Median (IQR)	23 (0, 106)	
Total Plaque Volume Median (IQR: 25–75), mm^3^	95 (39–200)	
Total Non-Calcified Plaque Volume Median (IQR: 25–75), mm^3^	56 (21–125)	

## Expert consensus approaches to plaque characterization versus AI-QCT

**Fig. 2:**
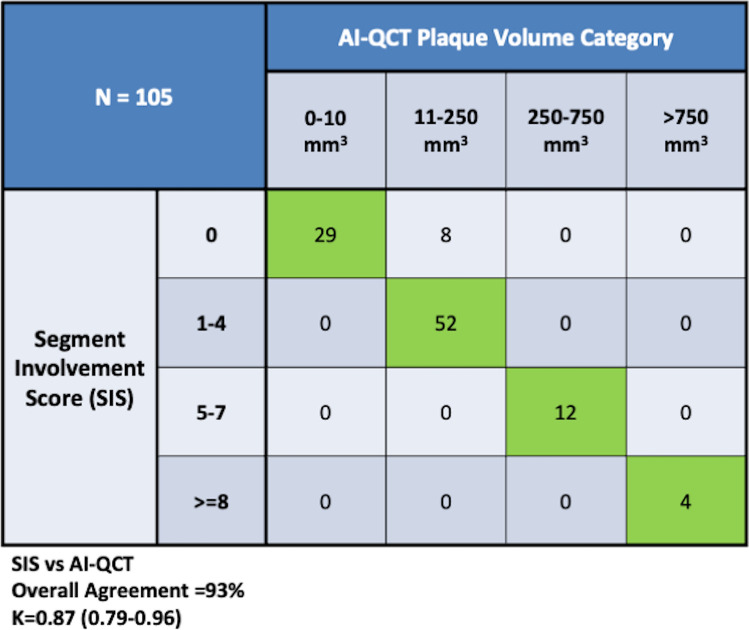
Contingency Table of SIS vs AI-QCT Plaque Volume. In comparing AI-QCT whole heart plaque quantification to SIS, there was high agreement with almost perfect reliability (93%; k = 0.87 [95% CI: 0.79–0.96]) as shown in Fig. 2. The AI-QCT was more sensitive to mild plaque burden (P1) than visual assessment of SIS by independent readers

Overall, there was high agreement (93%) between segment involvement score and AI-QCT plaque volume (Fig. 2). For 8 (8%) cases identified by SIS as having no plaque or AI-QCT measured 11-250 mm^3^ plaque burden. This would have changed the CAD-RADS 2.0 category from 0 to 1 with a P1 plaque classification. In discordant cases between AI-QCT and SIS, the TPV was smaller (median 53 mm^3^ [IQR 25–75: 37- 91]) vs concordant cases 98 mm^3^ (IQR 25–75: [39–205]; P = 0.001) as well as in non-calcified plaque volume that was smaller at median 29 mm^3^ (IQR 25–75: [28–61]) vs median 62 mm^3^ (IQR 25–75: [20–126]; p < 0.001).

In assessing visual estimate versus AI-QCT, agreement was modest at 64.4% (Fig. 2) with fair kappa classification = 0.488 (95% CI: 0.38–0.60). Among the 38 patients with no plaque by visual estimate 8/38 (21%) had quantitatively mild (11–250 mm^3^) and 1 had moderate (250–750 mm^3^) TPV. Among the 22 patients with moderate plaque by visual estimate, 18/22 (82%) had quantitatively mild (11–250 mm^3^) while only 4/22 (18%) had corresponding moderate visual and AI-QCT plaque volumes. In cases of concordance between visual plaque amount and AI-QCT, the total plaque volume was smaller with median 60 mm^3^ (IQR 25–75: [25–120]) when compared with cases of discordance with median 172 mm^3^ (IQR 25–75: [106, 251]; p < 0.001). Among patients with none or mild visual estimate of plaque, AI-QCT classified patients upward in 25% (25/105) of patients.

**Fig. 3:**
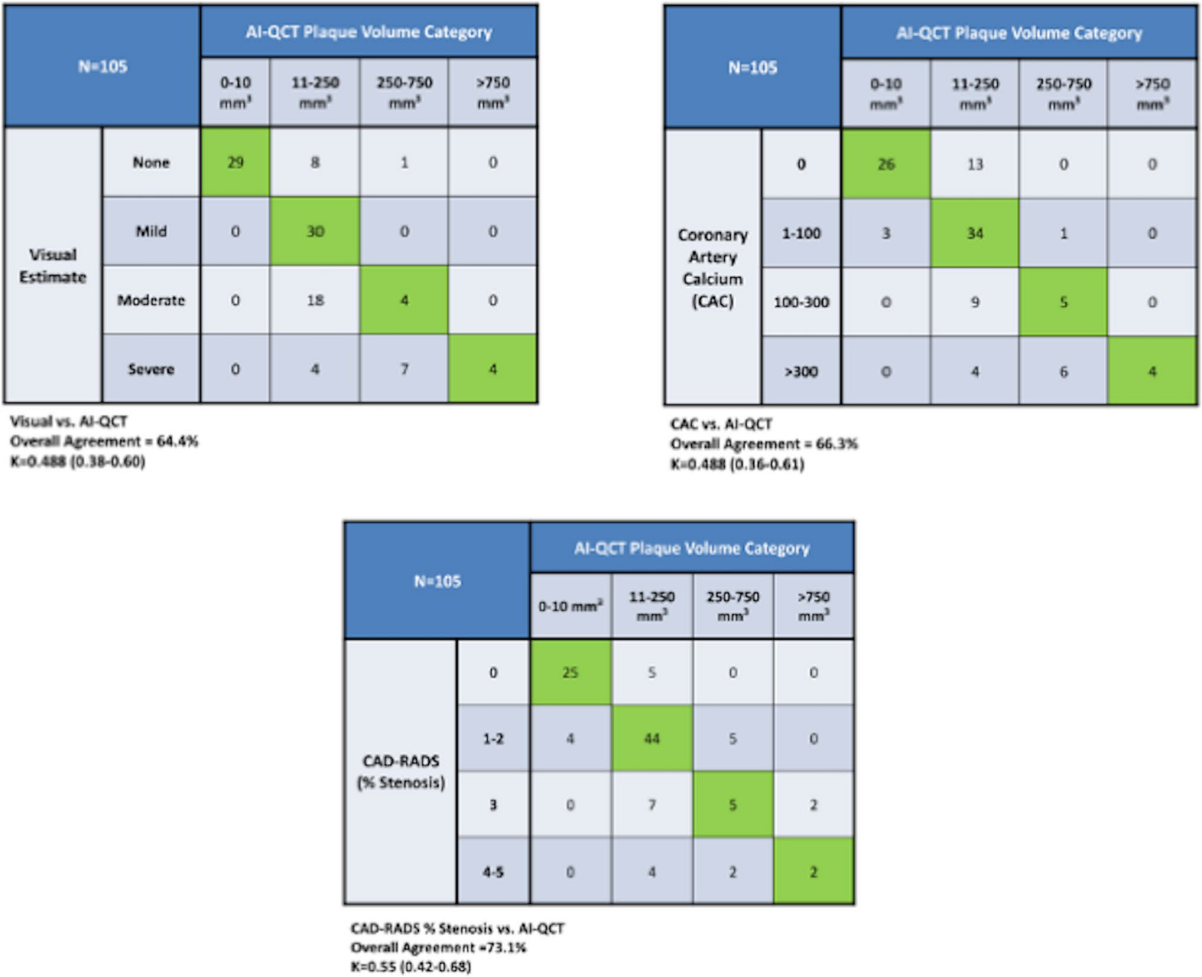
Contingency Tables of Visual Estimate, CAC and CAD-RADS % Stenosis vs AI-QCT Plaque Volume. Agreement between AI-QCT whole heart plaque quantification and visual assessment (64.4%; k = 0.488 [95% CI: 0.38–0.60]) and CAC (66.3%; k = 0.488; 95% CI 0.36 – 0.61) was fair. Agreement between AI-QCT and CAD-RADS % Stenosis (73.1%; k = 0.55; 95% CI: 0.42–0.68) was fair

Agreement between coronary artery calcium score and AI-QCT (Fig. 3) was also modest with fair kappa classification (66.3%; k = 0.488 [0.36 -0.61]). Notably, among the 39 patients with a zero coronary artery calcium score, 13/39 (33%) had demonstrable plaque composed of non-calcified plaque. In the discordant cases, total plaque volume was median 154 mm^3^ (IQR 25–75: [68, 245]) and non-calcified plaque volume was median 87 mm^3^ (IQR 25–75: [41–126]) which was higher than in the concordant cases TPV median 72 mm^3^ (IQR 25–75: [26–152]; p < 0.0001) and NPV median 37 mm^3^ (IQR 25–75: [14, 124]; p < 0.001). Among patients with 0 or mild (1–100) CAC, AI-QCT reclassified patients upward in 13% (14/105) patients through detection of non-calcified plaque.

Lastly, a comparison of AI-QCT versus CADS-RADS stenosis categories (Fig. 3) was moderate with overall agreement of 73.1% with a fair kappa classification of 0.55 (95% CI: 0.42–0.68). Among 30 patients with CAD-RADS 0 stenosis, 5/30 (17%) had demonstrable atherosclerosis by AI-QCT with TPV category of 11–250 mm^3^. Total plaque volume in discordant cases was higher with median 191 mm^3^ (IQR 25–75: [116–253]) when compared to concordant cases median 48 mm^3^(IQR 25–75: [25–96]; p < 0.0001).

**Fig. 4:**
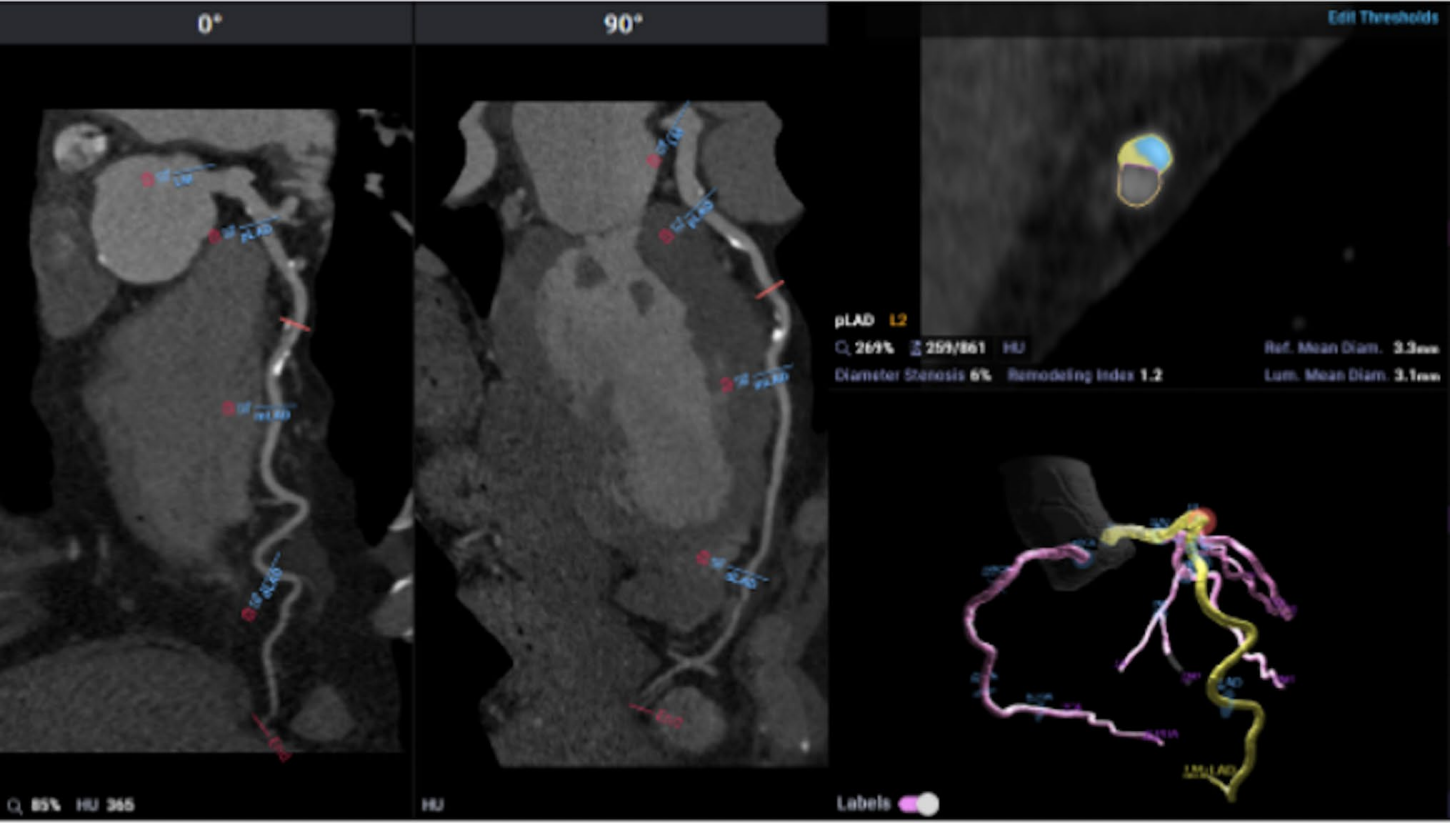
AI-QCT example of non-obstructive CAD that is concordant with SIS. Case example of a left anterior descending coronary artery in which plaque assessment by SIS and visual assessment was concordant with AI-QCT. The SIS is 4 with moderate calcified and noncalcified plaque burden involving the LAD on visual assessment. Applying CAD-RADS 2.0 categorization of plaque burden results in P2 moderate (SIS of 3–4 and 1–2 vessels with moderate plaque burden) category. AI-QCT was concordant with a total plaque burden of 567 mm^3^ which resulted in moderate (250–750 mm^3^) category

**Fig. 5:**
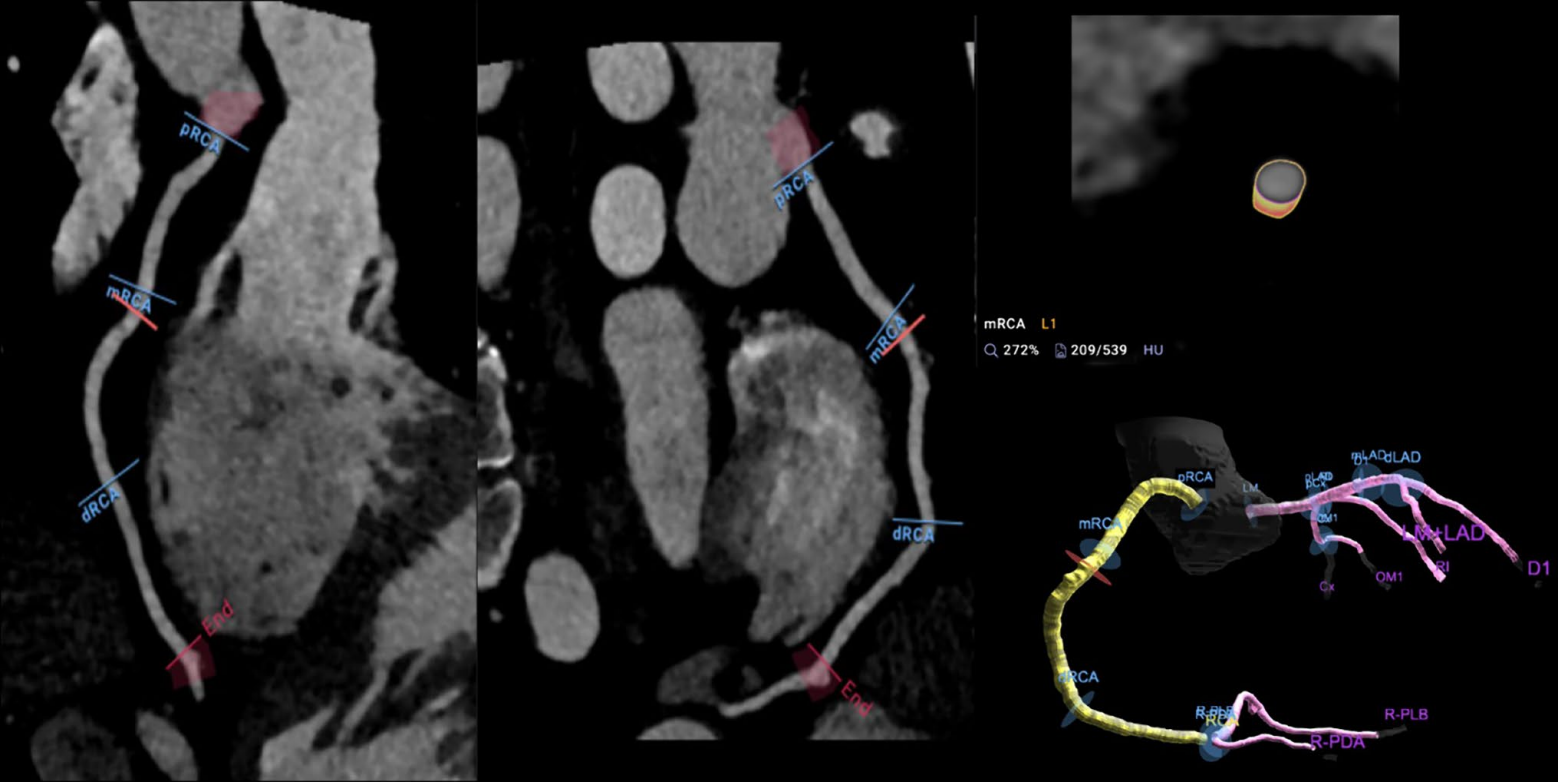
Non-Calcified Plaque detected by AI-QCT and missed by visual assessment. Case example of a right coronary artery with predominantly non-calcified plaque missed by visual assessment but detected by AI-QCT with plaque volume of 80 mm^3^. The resulting CAD-RADS 2.0 category would change from CAD-RADS 0 to CAD-RADS 1 with a P1 moniker (1 vessel with mild amount of plaque). Management changes would include referral for outpatient follow up with possible initiation of lifestyle modification and pharmacotherapy

Representative case example of SIS and visual assessment concordance with AI-QCT is shown in Fig. 4 while visual assessment and AI-QCT discordance is shown in Fig. 5**.** Applying CAD-RADS 2.0 plaque burden assessment to the case example in Fig. 4 there is concordant CAD-RADS 1 P2 with AI-QCT of 567 mm^3^ (moderate 250-750 mm^3^). In the example in Fig. 5; visual assessment, SIS or CAC score did not identify the subtle non-calcified plaque which would have resulted in CAD-RADS 0. AI-QCT detected the 80 mm^3^ non-calcified plaque and would have categorized the patient as CAD-RADS 1 P1.

## Discussion

This study provides novel data in comparing AI-QCT whole-heart plaque quantification to recently published multi-society expert consensus standards for assessment of atherosclerotic disease burden that include the SIS, CAC score, visual assessment and CAD-RADS % stenosis. Our analysis showed that there was moderate agreement between AI-QCT and CAC score (66.3%), visual assessment (64.4%), and moderate-agreement with CAD-RADS % stenosis scoring (73.1%). Finally, there was high agreement between AI-QCT and SIS in assessing and categorizing total plaque burden (93%). Discordance between AI-QCT and visual, CAC and CAD-RADS % stenosis was observed in higher total plaque volumes whereas discordance between AI-QCT and SIS was observed in smaller TPV and non-calcified PVs. The paradigm of atherosclerosis burden was recognized by the 2021 ACC/AHA multisociety Chest Pain Guideline by codifying the importance of identification of non-obstructive coronary artery disease [17]. This entity has been traditionally under-recognized and under treated as a means of cardiovascular prevention [5]. There are multiple recent trials that have found that plaque burden as assessed by CCTA is associated with a higher risk for cardiovascular events beyond stenosis alone [1, 3, 18]. Furthermore, it is well established that the majority of coronary lesions causing future MI are not from baseline severe angiographic stenosis [19–23]. Min, JK, et al. published the first large scale prognostic report examining the combination of stenosis, per-segment and per-vessel basis [4]. They found that having five segments with stenosis was associated with a 15% 1.5 year mortality. Synthesis of 17-published reports (n = 49,957) with median 2.5 years of follow-up found eightfold higher risk events among those patients with non-obstructive CAD [1]. The Western Denmark Heart Registry followed 23,759 patients for 4.3 years and found a stepwise increase in plaque burden with cardiovascular risk [3]. However this analysis was limited to coronary artery calcium burden and did not account for non-calcified plaque. While a CAC score provides a quantitative estimate of overall plaque burden, CCTA can also assess the burden of non-calcified plaque and uniquely allows for evaluation of adverse atherosclerotic plaque characteristics (APCs) or high risk plaque. These features include plaque burden and composition, and arterial remodeling; which demonstrate import for plaque instability and arterial wall injury.

Recognizing the importance of integrating plaque burden into structured reporting, CAD-RADS 2.0 added a moniker of “P” to categorize patients from P1 to P4 to describe the amount of plaque as mild, moderate, severe or extensive on a per-patient basis [7]. It was recognized by the CAD-RADS 2.0 writing group that inter-reader and inter-technique variability would exist; however, this concern was balanced by providing flexibility to institutions while trying to also minimize additional time or resources required to assess the plaque burden. The expert consensus recognized that providing flexibility would help ensure wide-spread adoption of reporting plaque burden in every CCTA report, and that ultimately even if small changes exist between techniques, ensuring that the “P-classification” – which was new in CAD RADS 2.0 –can be widely used was an important goal. The writing group also recognized that future iterations of CAD RADS would likely include novel techniques for plaque quantification as studies in this current report.

The addition of artificial intelligence guided plaque quantification represents an attractive approach to the assessment of plaque burden. AI-QCT PV accounts for total plaque volume including calcified and noncalcified plaque and is less prone to inter-reader variability in reader-defined semi-quantitative and visual methods. In the present study, while AI-QCT and SIS showed the highest agreement, discordance was observed at smaller plaque volumes that may be due to missed small volume plaque by clinician readers. The novel 4-stage system of staging patients used in this study was derived from the relationship of atherosclerosis plaque volume to ICA stenosis and ischemia in a large group of patients from a prospective multicenter clinical trial in which CCTA, ICA and FFR data was available [13]. Recent evidence has, furthermore, found that the total plaque volume categories evaluated in our study are associated with clinical outcomes with a stepwise increased risk of MACE [24].

It is not surprising to see that AI-QCT had limited agreement with manual-reader based standards of plaque characterization. CACS does not account for the totality of atherosclerotic burden as it does not include non-calcified plaque that may be further missed by visual assessment. Conversely, the present data suggests that there is categorical discordance at higher plaque volumes including the presence of non-calcified plaque.

## Limitations

There are several limitations that deserve closer attention beyond the small sample size in this single-center, single scanner post-hoc analysis. While this cohort was highly heterogeneous in ethnicity, the demographic characteristics of this urban cohort of patients that included half African-American and half women may not be fully representative of the entire population of patients such as Asian or Hispanic who receive CCTA scans. This study did not compare categorization of high risk plaque features such as low-attenuation plaque as categorization of these features is not part of CAD-RADS 2.0. Direct comparison of prognostication of CAD-RADS stenosis categories and plaque volume categories may not be fully comparable with the differences in prognostication yet to be established.

## Conclusion

Overall, AI-QCT showed moderate agreement with CACS and visual assessment, as well as moderate agreement with CAD-RADS % stenosis. While SIS and AI-QCT plaque volume staging had high categorical agreement, discordance arose where AI-QCT was able to detect even small quantities of plaque that SIS failed to detect. Our results demonstrate AI-QCT comparison to current expert consensus to effectively quantify and categorize plaque and improve future efforts for precise reporting of plaque burden, thus ultimately enhancing prevention and guiding future therapies. With ongoing validation, AI-QCT provides a rapid, reproducible, quantitative approach to total atherosclerotic burden assessment.

## Abbreviations

AI-QCT: Artificial Intelligence Quantitative Computed Tomography
SIS: Segment Involvement Score
CACS: Coronary Artery Calcium Scoring
CAD-RADS: Coronary Artery Disease (CAD) Reporting and Data System
CTA: Coronary Computed Tomography Angiography
MACE: Major Adverse Cardiac Events
SCCT: Society of Cardiovascular Computed Tomography
